# Homecare workers needs and experiences in end of life care: rapid review

**DOI:** 10.1136/spcare-2023-004737

**Published:** 2024-03-15

**Authors:** Catherine Forward, Zana Bayley, Liz Walker, Justine Krygier, Caroline White, Kasonde Mwaba, Helene Elliott-button, Paul Taylor, Miriam J Johnson

**Affiliations:** 1 Health and Social Care Workforce Research Unit, King's College London, London, UK; 2 University of Hull, Hull, UK; 3 Faculty of Health Sciences, University of Hull, Hull, Kingston Upon Hull, UK; 4 Wolfson Palliative Care Research Centre, Hull York Medical School, University of Hull, Hull, Kingston Upon Hull, UK; 5 Sheffield Centre for Health and Related Research, The University of Sheffield, Sheffield, UK

**Keywords:** Home Care Services, Social care, Terminal care

## Abstract

**Background:**

Social homecare workers provide essential care to those living at home at the end of life. In the context of a service experiencing difficulties in attracting and retaining staff, we have limited knowledge about the training, support needs and experiences of this group.

**Aim:**

To gain a timely understanding from the international literature of the experience, training and support needs of homecare workers providing end-of-life care.

**Methods:**

We conducted a rapid review and narrative synthesis using the recommendations of the Cochrane Rapid Reviews Methods Group. Building on a previous review, social homecare worker and end-of-life search terms were used to identify studies. Quality appraisal was conducted using a multimethods tool.

**Data sources:**

CINAHL and Medline databases (2011–2023; English language).

**Results:**

19 papers were included representing 2510 participants (91% women) providing new and deeper insights. Four themes were generated: (1) emotional support; homecare workers need to manage complex and distressing situations, navigating their own, their clients’ and clients’ family, emotions; (2) interaction with other social and healthcare workers; homecare workers are isolated from, and undervalued and poorly understood by the wider healthcare team; (3) training and support; recognising the deteriorating client, symptom management, practicalities around death, communications skills and supervision; (4) recognising good practice; examples of good practice exist but data regarding effectiveness or implementation of interventions are scant.

**Conclusions:**

Social homecare workers are essential for end-of-life care at home but are inadequately trained, often isolated and underappreciated. Our findings are important for policy-makers addressing this crucial challenge, and service providers in social and healthcare.

**WHAT IS ALREADY KNOWN ON THIS TOPIC**

- Social homecare workers form part of an essential care network and workforce that enables people to remain at home during the last months of life.
- Compared with other professionals delivering care in this context, they receive less training, support for career development or remuneration.

**WHAT THIS STUDY ADDS**

- Some training exists for homecare workers, but the most beneficial and acceptable content, delivery and implementation of training remains unknown.
- Homecare workers are often isolated from their own teams, and usually from the wider health and social care teams.

**HOW THIS STUDY MIGHT AFFECT RESEARCH, PRACTICE OR POLICY**

- Community-based healthcare and social care workers include social homecare workers in training, interdisciplinary communication and support regarding end-of-life care.
- The development and evaluation of widely available training content and guidelines for supportive practice are indicated to support this vital workforce.

## Introduction

 People living with advanced illnesses, approaching the end of life, often express a wish to remain at home as long as possible.^1^,^3^ Homecare provision is an essential part of the network of care, which can support them in this preference^4^,^6^ and is reflected in UK policy.^7^ With policy and practice changes, alongside an ageing population, there is likely to be an increasing demand for homecare services alongside an increasingly complex client base with comorbidities requiring end-of-life care.^8^

Social homecare workers not only support people at home with activities of daily living, but also provide emotional support and can help avoid unnecessary hospital admissions or unwanted transfer to institutional care.^4^,^6^ Despite their essential role, homecare workers remain poorly paid, often lacking the support and training they need to fulfil a range of caring roles when clients have such complex needs.^9^ Homecare agencies in the UK and elsewhere are struggling to recruit and retain care staff; a chronic problem exacerbated in the UK by the departure from the European Union and the effects of the COVID-19 pandemic.^10^,^12^ Concerns around the difficulties of attracting people to the care workforce have focused attention on aspects of their working environment such as training and support.

The evidence around the experiences and training needs of homecare workers is limited. A review conducted in 2013 showed only nine papers examining the role of support workers in providing end-of-life care, eight of which studied assistant healthcare workers and only one examined social homecare workers.^13^ However, the available literature confirms challenges regarding a lack of training about the specific needs of this client group, the emotional labour involved in this care and a lack of support for the homecare worker as health and care needs of their clients change.^13^,^16^ Given the ongoing difficulties in recruiting and retaining care staff,^8 17^ a more comprehensive understanding is required of the specific training and support needs of homecare workers when providing end-of-life care.

With a better understanding of the needs and experiences of homecare workers, more effective training and support can be developed to improve working conditions and care delivery. This review provides an update of the 2013 review^13^ and focusses on the experience of homecare workers, as distinct from healthcare practitioners, given their different role, training background and working environment.

## Methods

### Design

Given the focused nature of this topic and the current crisis in social care provision in many countries, a rapid scoping review was considered the most suitable to gain an insight into the current availability, content and quality of evidence.^17 18^ Rapid reviews are commonly used when an understanding of the current evidence regarding a topic is required and resources, such as time, are limited. Quality searching and reporting standards still apply to ensure that the results remain relevant and reflect an accurate representation of the evidence.^18^ We conducted this narrative rapid review guided by the recommendations of the Cochrane Rapid Reviews Methods Group.^19^ We report it in accordance with the Preferred Reporting Items for Systematic Reviews and Meta-Analyses (PRISMA) statement.^20^ The review was not registered, and a protocol was not prepared.

### Search strategy and study selection

To build on the work by Herber and Johnston,^13^ we used the same search terms for homecare workers, but excluded any related to healthcare workers (see table 1). The journal databases Medline and CINAHL were searched from May 2011 (the end date of the original search) to March 2023, restricted to English language texts and peer-reviewed papers. Given the topic and paucity of the literature, there was no restriction on the type of studies. The search was updated on 5 December 2023

**Table 1:** Search terms

String #1 terms for staff group (Boolean OR)	String #2 terms for setting (Boolean OR)	String #3 terms for patient group (Boolean OR)
Ancillary staff OR Care assistant OR care support worker OR care workers OR CNA OR community support worker OR domiciliary care assistants OR home helper OR home-care support worker OR home-care worker OR in-home supportive services OR LPN OR social carer OR unqualified aids	Care in the community OR Home OR home care OR home-based care	Life-limiting illness OR terminally ill OR end-of-life OR palliative OR dying

**Figure 1:**
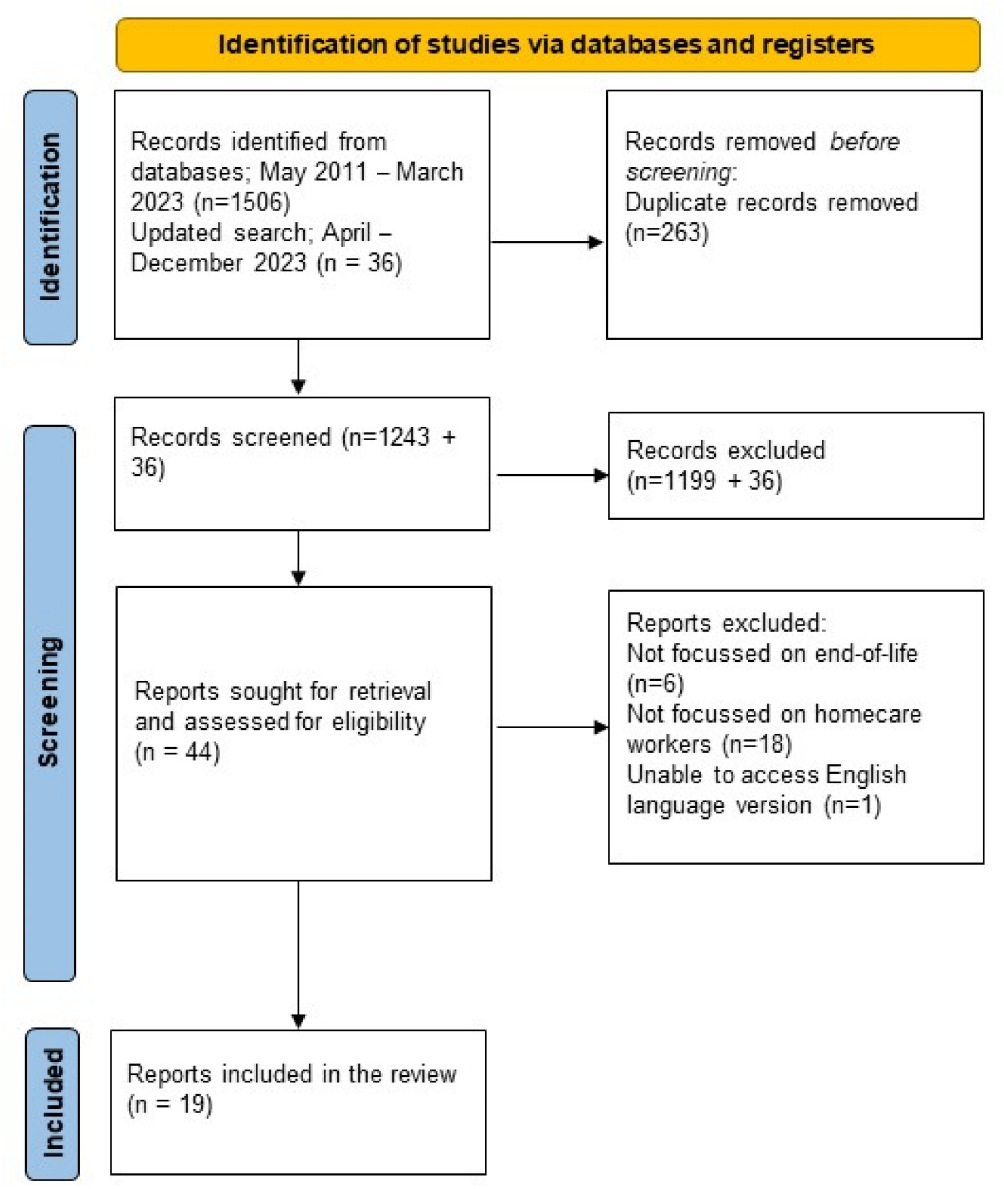
Preferred Reporting Items for Systematic Reviews and Meta-Analyses flow diagram.

Retrieved titles and abstracts were screened initially by one researcher (CF) against a priori eligibility criteria (see table 2) and checked independently by at least one other researcher (KM, ZB or JK). An additional reviewer (MJJ) was available in the event of persistent disagreement. In cases where it was unclear if a paper was suitable for inclusion, full papers were retrieved for further assessment for inclusion. Rationale for excluding papers included the population (eg, not end-of-life clients or patients) and the setting (eg, not homecare). The full process and reasons for exclusions are detailed in figure 1.

**Table 2:** Inclusion and exclusion criteria

Inclusion	Exclusion
Having insight into the needs or experiences of homecare staff working with those at end-of-life or with life-limiting illnesses.Peer-reviewed publications.English language papers.	Focus on other professions such as healthcare workers.Not in the domiciliary (home) setting.Literature reviews.Duplicating findings from other papers.

#### Data extraction

Data from included studies were extracted using a customised extraction form to record the study setting, focus and aims, study design, methodology, country of origin, client group and summary of findings. Data were extracted by author CF and independently checked by ZB and KM.

Quality appraisal was conducted using a tool (Hawker *et al* designed to apply to different study designs^21^) used in the original Herber *et al* review^13^ by CF. Papers were independently checked by one other researcher (ZB or JK) with any discrepancies being resolved by an additional researcher (MJJ).

#### Data analysis

A narrative synthesis^22^ of the characteristics and key findings of the studies was undertaken. The key findings from each included study were initially summarised by CF and patterns observed to form a preliminary synthesis. This allowed a textual overview of the key issues. Relationships within and between studies were then explored, including possible explanation, which were discussed by all authors and revised iteratively to agree finalised themes.

## Results

### Selected studies

The first database search identified 1506 papers; 1243 following deduplication. The process of selection is shown in the PRISMA flow chart (figure 1). Following screening, 19 papers were included (see 1online supplemental table 1). Two papers tested the same training evaluation; however, they focus on different aspects and therefore both are included in the review. No studies were identified from the updated search.

### Characteristics of selected studies

#### Study design

Eight qualitative,^523^,^29^ six quantitative papers^30^,^35^ and five mixed methods studies^1536^,^39^ were included. Qualitative data collection was primarily collected through interviews,^5 23 26 29 39^ focus groups^15 24 27 36 38^ or a mixture of interviews and focus groups.^24 28^

The quantitative studies were observational, with data collected using questionnaires or surveys^1530 33^,^35 38^ and two reports from one randomised controlled trial.^31 32^

#### Study aims

Three papers evaluated training delivered to either homecare workers^15^ or multidisciplinary teams that included homecare workers.^31 32^ These latter two papers reported on the same training evaluation; however, they focused on different aspects of the training and therefore are both included in the review.

Seven of the papers sought to explore the experiences of homecare workers working with clients at the end of life.^523^,^28 30^ This included considering their challenges, potential training and support needs as well as reflections on good practice of homecare workers when working with those with life-limiting illnesses. Three studies had built on earlier work to develop training materials for homecare workers. Several papers reported findings from the impact or evaluation of these materials and training programmes.^15 31 32 38^ In addition to training, there were other studies that aimed to improve support for homecare workers. One such paper included a symptom assessment solution, which aimed to help staff assess changes in symptoms and any associated decision-making, aiming to increase skills and confidence.^25^ This study used focus groups to plan such a programme but was not evaluated. Another study explored the nature and importance of effective communication on the relationships established between non-clinical workers and service users.^39^

#### Setting

Included papers came from predominantly ‘Western’ nations and Japan (see online supplemental table 1). Five papers were from the UK,^5 15 24 26 29^ four from the USA,^25 27 28 36^ two from Canada,^23 37^ one from Australia,^38^ one from Sweden,^30^ and five from Japan^31^,^35^ and one from Hong Kong and Australia.^39^

#### Participants

In total, 2510 participants are represented. In those studies which reported data on gender, 91% of participants were women.

1261 research participants were homecare workers and managers, while 1249 were other stakeholders such as nurses, doctors, clients, family carers or other professionals, although the level of data reported means that the percentages of each group are not calculable. Some studies were a mix of homecare workers and health and social care professionals such as District Nurses or Local Authority managers (n=6^24 25 31 32 35 39^), or gathered data from the homecare agencies more generally (n=2^33 37^). Two studies included families of those receiving homecare, although recruitment from this group was understandably difficult and limited.^26 38^

While the focus of this review was on homecare provision around the end of life, the papers varied in terms of their patient group focus. 12 papers considered end-of-life care for people with a range of conditions.^1526 28 30^,^35 37^ Seven of the papers focused on specific conditions (dementia, n=5^5 23 24 27 29^; heart failure, n=2^27 36^), while another focused on symptom management such as shortness of breath or pain in a range of illnesses.^25^

### Findings

The narrative analysis is presented in four themes that are evident across the literature: emotional support; interactions with other practitioners; training and support needs; and recognising good practice.

#### Emotional support

While the commissioned role undertaken by homecare workers for end-of-life care is usually practical provision of personal hygiene or medication support, much of the described support to people with advanced conditions towards the end of life can be more complex, involving supporting the client and their family with more intangible emotional support:

They [clients, families] don’t know what’s going on, they don’t know what to ask you, they don’t know what to expect of you […] you walk into this environment of … it can be chaotic and other dynamics are … you can sense it as soon as you walk in. There’s a small degree of panic, […] but they are, I have to say overall, very, very, very grateful of anything that you do. (community care worker 8) [38, p275]

There is significant and nuanced emotional labour in supporting a client and their family through this period of life.^24 25 27 28^ This is aggravated by the isolating and isolated nature of homecare work leading to staff feeling undersupported and underprepared. Unlike healthcare workers, social homecare workers rarely have explicit and structured support such as an equivalent to clinical supervision. Support is required to reduce the impact of the emotional and cognitive stresses of working so closely with those at end of life, and the associated grief, to reduce the impact of these on staff and to limit stress and burnout.^27^,^29^ Both peer and manager support were suggested as useful resources to help reduce such stresses, but can often be unstructured and not embedded in formal processes:

“It’s quite ad hoc if I am honest. We do not have like a, you know, a person, we do not have like a, what do you call them, a therapist or a counsellor or anything for people to go to. I think any of the office staff here would always listen and lend an ear to the care workers.” (male homecare worker, aged between 25–40). [29 p357].

The widespread impact of the COVID-19 pandemic brought the need for additional support, and key areas of practice which require improvement such as communication skills—both verbal and non-verbal—into sharp focus.^30^ However, there was limited detail on what this should look like and how the effects of grief and loss might be mitigated or how such support would be implemented in practice in the context of understaffing and limited financial resources.

#### Interaction with other practitioners

The changing nature of providing care for clients with progressive, advanced conditions and their families is a particular challenge in the context of end-of-life care.^5 35^ Given the complex and changing situation, the wide team of practitioners, family and friends involved in the client’s care, communication between other care providers (both professional and informal) and homecare workers was difficult and aggravated by the lack of systematic communication between them.^34 38^ Homecare workers reported a lack of clarity regarding their role:

“The agency doesn’t make clear to anyone who we are or what we do. You know, we’re not just the help!” [27 p4]

The lack of clarity, particularly among the wider healthcare team, left homecare workers feeling poorly understood and valued and led to them being left out of wider support and communications:

‘Because you’re a carer you get pushed to the side and people don’t listen to what you’re saying but when we come to [the hospice] nobody does that, and people can ask the things that they’ve been afraid to ask. And having the professionals the other side has helped to answer some of the questions that we’ve not always been able to get the answer for’. [15 p26].

“We’re with the clients all day, more than anyone else. So why aren’t we included in the conversation?” [38 p5].

An evaluation of an educational booklet used as a basis for focus group discussions aiming to address poor interdisciplinary understanding and working in the wider social and healthcare team notably improved mutual respect and appreciation.^30^

In contrast to the isolated homecare worker, community palliative and primary care teams are well established and multidisciplinary in nature. Unqualified healthcare workers practice in an environment which has a comparatively well-established training and support system and often work closely with qualified healthcare professionals. Homecare workers report working with less support and training than their healthcare colleagues. Homecare workers practice outside of the healthcare structure and yet are expected to work with an increasingly complex client group, often outside of ‘normal’ working hours (with little access to advice from other service workers) and alongside the additional support services which accompany this:

“You’re calling and calling. You’re by yourself and no one picks up at the agency. You’re stranded with the client, and you don’t know what to do and you need help. It’s a huge problem.” [38, p5].

#### Training and support needs

The need for specific training support in the end-of-life context was clearly recognised alongside the current gap in provision for homecare workers. Four papers presented findings from training programme evaluations for homecare workers.^30 31 37^ The findings identified that the content and delivery of training for homecare workers, including communication skills training (verbal and non-verbal) requires further work to establish the optimum training content and to evaluate the outcomes in staff learning, confidence and practice. There was an indication that homecare workers would benefit from increased knowledge to help them access support for their client when they deteriorate:

You see a change, but you can't go on the phone and say to the doctor, ‘Well she’s changed,’ because there isn't a word for it […] it’s like a gut feeling for me. [Staff focus group, manager] [24, p5].

Condition-specific literature also highlighted the need for focused training around challenging symptoms associated with dementia or heart failure,^5 35^ and the importance of understanding policies and procedures for after a death has occurred for staff confidence:

You need to know that these are the steps that you have to follow, like reporting death policy that you would have in place, so you know what to follow. (female homecare worker, aged between 18 and 24). {5 p1988].

While many examples of good practice were described, the homecare workforce is often underprepared for the complexity of caring for those who are approaching the last months of life, despite being a key service provider in this situation. Some evaluations of training initiatives reflected increased confidence at work although this did not translate to increased job satisfaction; further development and testing of training programmes is needed to evaluate changes in practice and client experience and outcomes.^15 30 31^

A suggestion that less experienced staff would benefit most from training reflected an assumption that staff learn skills and confidence ‘on the job’, further emphasising both a gap in current practice and the nature of the knowledge required.^29^ While training provision was clearly a positive aspect, barriers to training were found regarding consistent attendance at training sessions,^15^ suggesting that while the establishment of a training programme is laudable, the implementation of this must consider the challenges of working with a busy, community-based workforce and the costs of provision and/or access. Condition-specific training was also identified as a need,^22 35^ as the presentation and symptoms of different conditions require different management.

#### Recognising good practice

Examples of good practice included recognition of, and communication that, a client may be approaching the last months of life; something which can be difficult in chronic conditions with which clients have lived for many years.^23^ Other good practices included regular scheduled supervision-type processes:

There is good sides, but there is often especially if you look after someone with dementia, there is also a lot of stress. And so, what we have done to help reduce that stress is every month we have one of the directors who has (a faith-based background). This person does reflective supervision with the staff (P25, male homecare manager, aged between 25–40). [29, p 357].

Homecare workers may have provided care for clients with long-term conditions who are now reaching the end-stage of disease, but the homecare worker is unaware of this change. Good communication between homecare and health professionals, clients and their informal carers is a consistently recognised factor in good practice, although, with limited evidence as to how this might be operationalised. A good understanding, and appreciation, of the role of each professional was noted to improve collaboration, with interprofessional communication helping to provide better quality care for clients.^34^ The changing nature of end-of-life care was reflected in several of the papers, as conditions, symptoms and function can fluctuate unpredictably, creating stress for the client, their family and the homecare worker. The findings suggest that this is approached with flexibility on the part of the homecare worker, however, having the confidence and skillset to be able to do this is something which requires experience and training.^5^

#### Summary findings

There are significant support and training needs regarding end-of-life care provision in domestic settings such as the need for communication and mutual role clarification and respect within the wider multidisciplinary team, and further training on managing changing function or symptoms, and what to do after a death. Condition-specific literature highlighted the need for more focused training, especially around challenging symptoms and behaviours associated with common diagnoses such as dementia or heart failure. Also indicated is a need for training homecare workers who may require additional support around both verbal and non-verbal communication skills. Finally, increased support is required around the emotional and cognitive stresses of working so closely with those at end of life, and the grief and loss associated with this.

#### Quality appraisal

The quality appraisal found a generally high level of quality, including in the observational studies where there were robust sampling methods and confounders were addressed in the analysis. Online supplemental table 2 shows the scoring for each of the papers included in the review. The most common omissions were a lack of reporting on bias^24 25 37 38^ and limited samples such as from one organisation^15^ or homecare workers from a particular union.^26^ Other omissions were reporting ethnicity or other demographic information,^23 36^ which might have helped indicate the generalisability of the findings.

## Discussion

Homecare workers caring for those at end of life provide care in the context of complex, changing and often emotionally charged situations. They care for clients with advanced and progressing disease, with little to no specific training on managing changing physical function and symptoms, client and family distress, or on verbal and non-verbal communication. Navigating the personal–professional boundaries which may become blurred with a dying client requires skills and good supervision and support which is commonly lacking. Working alongside, but employed outside of, a network of healthcare practitioners and family and friends, means that there is a need for clear and reliable communication within the wider multidisciplinary team. Failure to do so aggravates the emotional burden experienced by homecare workers if; (1) their role is not clear to themselves or others, (2) their role is unappreciated by the extended team and (3) they are unaware of other practitioners’ input, support or clinical care plans relating to deteriorating health. Being faced with unmet need of a client with whom the homecare worker may have developed a strong relationship, homecare workers may ‘self-extend’ their duties beyond their skills, training, responsibilities and capabilities. Homecare workers remain isolated and expected to learn through experience. The need for better training and support is recognised, but there is little information as to the content, the effectiveness or implementation of these important interventions in practice.

Our review highlights the emotional and psychological impact of caring for those approaching the last months of life. It indicated the need for additional support to reduce the impact on staff and limit stress and burnout^27^,^29^ but provided little detail about what this should include, or how it should be provided. The isolating nature of homecare work was seen particularly through the COVID-19 pandemic with calls for additional support.^32^ The emotional support needs of homecare workers working within end-of-life care are recognised in the wider literature.^39 40^ The distress experienced by care workers likely contribute to the issues around staff retention and staff absence in this service industry.^28^ Both healthcare and social care services include workers with no formal qualifications, in addition to registered professions such as nurses or therapists or social workers. In some publications, these unqualified groups are treated as synonymous,^13^ but in practice they have different training and support networks. Within the context of end-of-life healthcare, it is recognised that caring for those at end of life requires specialist skills and knowledge. Community palliative care and primary care teams are well established and are multidisciplinary in nature, with each profession having specified training and supervision requirements, and unqualified and qualified healthcare staff often work closely together. By contrast, homecare workers appear to work with less support.^9 10^ Of note, a similar experience can also affect healthworkers—particularly the unqualified healthcare assistants—who, although being part of the multidisciplinary healthcare team, in practice work alone.^41 42^

In healthcare settings, there is an evidence base regarding staff well-being, for example, in palliative care settings, acknowledging the level of emotional labour in this staff group and increased risk of burnout compared with other settings.^39 42 43^ Interventions include meditation, arts-based supervision practice and improving communication skills all with the aim of addressing the emotional support needs of those working in palliative care.^43^ Other examples of addressing this element of emotional labour include Acceptance and Commitment Therapy,^43^ orientation or induction programmes, access to bereavement advisors, team debriefs and peer support.^44^ An emerging literature shows that clinical supervision for healthcare workers in general is associated with better well-being,^45^ lower rates of burnout and better staff retention.^46^ However, the recognition that supervision is often the first aspect of work to be lost under increased work pressures, such as during the pandemic^46^ means that implementing this into the already pressured world of homecare would be challenging.

Our review highlighted the importance of communication with the wider team involved in the care of clients at the end of life. They are present in the home with the client and their families and face difficult conversations and potentially increased risk if they are not fully included in healthcare communications.^47 48^ However, not only does the homecare worker need to be informed by the healthcare team of advance care plans and changes in care goals, but they also know their clients well and may be the first to recognise deterioration or a change in need. Effective three-way communication (written and verbal) among the client, carers and healthcare professionals could improve appropriate escalation and avoid unnecessary hospital admissions.^48^,^51^ In addition to recommending increased training in communication for homecare workers, this review indicates that healthcare workers would also benefit from training about social homecare worker roles and skills and the importance of including homecare workers in communications and what a homecare worker’s role entails. A fundamental shift in the respect and appreciation between the whole social and healthcare team would foster better teamworking and homecare worker confidence and support. The shift in the UK to electronic documentation for homecare workers may help support more effective communication if utilised effectively, but further evidence on this is required.^3^ There is a current drive in the UK to integrate health and social care services,^52^ citing the community multidisciplinary team as a potential mechanism to improve care.^53^ However, while social workers may be members of such teams, homecare workers and their managers are not.

We identified a current gap in training and support for homecare workers in respect of end-of-life care. While many examples of good practice were described, the homecare workforce is underprepared for the complexity of caring for those who are approaching the last months of life, despite being one of the central professions involved. The training initiative evaluations included in our review showed an increase in confidence in their work but not in job satisfaction indicating the need for further development and testing to show changes in practice.^30^ The suggestion that less experienced staff would benefit from training reflected the tendency for staff to learn skills and confidence ‘on the job,’ highlighting both a gap in current practice and the nature of the knowledge required.^29^ The assumption that less educated workers need little formal training and that ‘on the job’ experience over time is sufficient, is a pervasive problem recognised across many fields of employment, and a persistent training gap lies with a lack of supply of training by the employer rather than a low demand by employees.^54^

While training provision was seen as positive, barriers to training were highlighted particularly in terms of ability to attend.^15^ The implementation of a training programme must take the challenges of working with a busy, time-poor community-based workforce into account. We found some evidence of good practice but there does not appear to be a national or international standards for homecare at the end of life. A rapid scoping review of systematic reviews, focussing on service delivery models to maximise quality of life for older people at end of life, found limited reference to social care.^55^ A King’s Fund report on homecare in England made no reference to palliative or end-of-life care,^56^ similarly for the 2020 Skills for Care annual review of adult social care.^57^ Further, in England, the Government policy vision for adult social care makes minimal reference to the need for/provision of support at end of life.^58^

### Strengths and limitations of the review

A rapid review method inevitably limits depth and rigour compared with a systematic review. For example, no grey literature or non-English publications were included which may have yielded further insights into the needs and experiences of homecare workers. However, we followed standard guidance for rapid reviews.^19^ The inclusion and exclusion criteria and search terms were agreed a priori and the data extraction was carried out systematically and in discussion with the research team. However, we acknowledge that there are a number of terms used to describe social homecare workers, some of which are hard to distinguish from healthcare; therefore, we may have excluded some relevant papers. A formal quality appraisal of the papers was carried out using objective and transparent processes,^21^ showing the papers to be of good quality, enabling us to have confidence in the findings.

Compared with Herber and Johnston,^13^ we found significantly more published literature about social homecare workers (a further 19 papers, vs 1). This indicates a growing interest in this issue, but still highlights a lack of work evaluating solutions to the challenges; most were qualitative or observational in nature. We were able to confirm previous findings including applicable findings from the healthcare assistant population.^13^ We gained new and deeper understanding particularly regarding the isolation from the wider multidisciplinary team involved in the care of the dying person, lack of role clarity, lack role respect and understanding by the wider team and potential benefits from educational and team-working interventions. However, despite the increased literature, there were no studies from countries other than ‘Western’ nations or Japan. The focused nature of our review question means that other models of homecare provision, for example, the community volunteer service in Kerala, India,^59^ is out of scope.

### Implications for policy, practice and research

Social care needs of older adults are expected to double in England by 2040 and those requiring 24-hour care will rise by over a third by 2035.^60^ Homecare services, provided mainly by private agencies, are increasing in the UK, although with considerable turnover, with the adult social care workforce making up 5.3% of the English workforce (1.47 million employees).^4 57^ A large excess in home deaths was seen throughout the COVID-19 pandemic.^61^ District nurses and homecare workers provide the backbone of homecare. The lack of integration and strain on both services leaves end-of-life care at risk in face of a projected 42% increase in demand by 2040.^62^ This provides context to a wider need to consider structural factors such as the socioeconomic position of homecare workers, the recognised need for the increased professionalisation of this sector and the value of care labour more generally.^9^

Our findings are widely applicable in a ‘Western’ context and highlight several key areas for consideration for future policy, research and practice including training and support needs, emotional support and recognising good practice.^18^

An understanding of homecare workers’ education and support needs and how they integrate with the wider healthcare workforce is vital for the White Paper call for integrated care systems.^56^ In addition, a Health Foundation briefing document^63^ described how ‘social care issues are under the radar and underappreciated. The longstanding political neglect of social care in England has been laid bare for all to see. Continued neglect would leave the system vulnerable to future shocks’. Despite social care reform being a stated UK top priority with the COVID-19 pandemic pushing it ‘up the political agenda and on to the front pages’,^64^ to date, successive governments have failed to materially address these failings in policy change supported with necessary funding.

Caring for those approaching the end of life in a home environment requires practices which cannot only improve the quality of care for the client but also the experience of those caring for them—both paid and unpaid. Notable features of good practice include a recognition and communication that a client may be approaching the last months of life, something which can be difficult to establish with confidence in chronic conditions which clients have lived with for many years.^23^ Good communication between care and health professionals, and with the client and their unpaid carers, is a consistent factor in good practice, although, there is limited evidence as to how this might be operationalised, demonstrating that flexibility is something which requires experience and training.^5^

Training needs are identified across a range of topics such as communication skills, multidisciplinary working, symptom management and managing the emotional labour involved in caring in this context. However, further evidence is required to inform the content and nature of this training to make it effective and accessible. This review indicates that the role of the homecare worker providing care to those approaching the end of life is one which requires flexibility and effective communication. In practice this means that both homecare workers and their colleagues in the wider multidisciplinary team should be aware of the need for good communication by discussing changes in care needs, function or advanced care planning. Those responsible for supporting homecare workers should be aware of the complexity of caring in this context and provide both practical and emotional support within the limits of the existing evidence.

With regard to future research, a knowledge of the overall priorities within the sector, and careful consideration of the effectiveness, delivery and implementation of training or other interventions are priorities. Training within work time or paid attendance needs to be considered otherwise the uptake will be limited especially as there is no current career progression attached to such training. A better understanding is required of what would make future training relevant and accessible to improve uptake, including a focus on content (such as communication skills, legal aspects of end of life, managing emotions and accessing support, working with the wider team) and the optimal forms of delivery (such as face to face or online). Training resources built on examples of good practice, codesigned with homecare workers and informed by their stated needs, are required. Training for those working alongside homecare workers, such as community palliative care teams, should include content about the role of homecare workers to promote good quality interprofessional communication across health and social care boundaries. Lastly, we found no studies regarding paid homecare support at the end of life in low-middle-income countries and non-‘Westernised’ cultures. This is important as the ageing population in many Asian countries, and changing culture with more women in paid employment, will lead to challenging practical implications on the provision of homecare by family at the end-of-life.^65^

## Conclusion

Homecare workers provide essential care to enable end-of-life care at home, but need further training and support, and recognition by and inclusion with the wider healthcare team. While some training has been developed, there is little evidence of widespread adoption of end-of-life training and effectiveness, and implementation evidence is scant. Our findings are important reading for policy-makers attempting to address this current and crucial challenge, and service providers in both social and healthcare.

## supplementary material

10.1136/spcare-2023-004737 online supplemental file 1

## Data Availability

All data relevant to the study are included in the article or uploaded as online supplemental information.
