# Supplementary material for: Homecare workers needs and experiences in end of life care: rapid review
Source: BMJ Support Palliat Care. 2024 Mar 15;14(e3):e004737. doi: 10.1136/spcare-2023-004737 (PMC11671894; doi:10.1136/spcare-2023-004737)
Supplement: online supplemental file 1 [file spcare-14-e3-s001.pdf]

Online Supplement Table 1. Characteristics of included studies, with quality appraisal score

Author (date) Country	N (gender)	Setting	Role title	Study aim	Method	Findings	Quality *
**Abrams et al (2018) UK [5]**	42	Community setting	Homecare workers & managers	To explore the need for flexibility when negotiating professional boundaries in the context of home care, dementia and EoL	Semi-structured interviews	Boundaries, can be altered in dementia care, particularly at EoL	35
**Baik et al (2021) USA [36]**	41	Non-profit training and education organisation	Homecare workers	To elicit educational needs and priorities of workers caring for community-dwelling adults with heart failure at EoL	Nominal group technique - semi-quantitative structured group process and point rating system	There is a need to develop and evaluate an educational program for workers working with people with heart failure at EoL	36
**Breen et al (2021) Canada [23]**	15 (14 female)	Community setting	Personal support workers	To explore the perceptions of workers regarding what constitutes quality home care for persons with dementia.	Semi-structured interviews	Workers require increased support through ongoing dementia-specific education and training, increased teamwork and wages, sufficient and qualified staffing, and increased client information.	35
**Craftman et al (2022) Sweden [30]**	122 (118 female)	Community setting	Homecare assistants	To describe workers' attitudes towards the care of dying persons living in their own homes.	Cross-sectional study	Need for increased training. Challenges are around communication, emotional attachment and caring for those dying.	34
**Fujita et al (2019) Japan [31]**	291	Community setting	Home care nurses, care managers, heads of care workers	Evaluation of the elements of interprofessional education for end-of-life care among homecare nurses, care managers, and head care workers	Cluster-randomized clinical trial	Care managers and head care workers had better understanding of own and other professional roles in EoL care, and better confidence in collaboration among health and welfare	31

						professionals. No changes observed for nurses.	
**Fukui et al (2019) Japan [32]**	291	Community setting	Home care nurses, care managers, heads of care workers	To assess the effect of a multidisciplinary end-of-life educational intervention program on confidence in inter-professional collaboration and job satisfaction among health and social care professionals	Cluster randomised control trial	For care managers and head care workers, their confidence improved with training but no significant change in job satisfaction.	33
**Igarashi et al (2015) Japan [33]**	1,097	Community setting	Homecare nurses, home helpers & care managers	To clarify institutional factors associated with EoL	Cross-sectional survey	Care agency level (e.g., size, preparedness) make it more likely that they will have EoL clients.	32
**Lee et al (2017) UK [24]**	87	Community setting	Service managers & frontline staff	To explore the views of service managers and frontline care staff on key aspects of good EoL care for people with dementia	Focus groups and interviews	Themes: Recognising EoL and tools to support EoL care; Communicating with families; Collaborative working; Continuity of care; Ensuring comfort at EoL; Supporting families; Developing and supporting staff.	33
**Manson et al (2020) England [15]**	80	Community setting	Home care workers	To test the acceptability of Project ECHO to workers as a way to improve knowledge of, and confidence in, delivering palliative care, and its effectiveness in reducing isolation by developing a community of practice.	Mixed-methods service evaluation	Project increased self-reported knowledge and confidence. Attendance was variable.	29
**McPherson et al (2019) Canada**	14 (all female)	Community health setting &	Unregulated care providers	To identify the types and frequencies of tasks performed by workers in	An exploratory two-phase sequential mixed method design	Challenging and complicated role, especially in the home	35

**[37]**		Community care setting		home-based palliative care to older clients		setting. Tasks often include emotional support.	
**Odierna et al (2018) USA [25]**	50 (34 female)	Community setting	Administrators, case managers, home service providers & consumers	To explore whether and how to integrate symptom assessment into an IHSS program to identify and manage symptoms in diverse older adults who receive in-home care.	Qualitative study comprising 10 semi structured focus groups	A symptom assessment program is desired, needed, and feasible and can leverage the established service infrastructure and relationships of consumers and service providers to assess symptoms in the home.	35
**Percival et al (2013) UK [26]**	42	Community setting	Home care workers, patient and family members	Study of the worker role in meeting the needs of families caring for those dying at home	Qualitative formal interviews	Specialist care workers had more time and expertise to give care needed (compared with usual homecare workers).	33
**Poulos et al (2017) Australia [38]**	39 (11 female)	Community setting	Family carers, community care workers & community palliative care team practitioners	To examine whether specially trained community care workers effectively support patients and their families in the home setting at EoL?	Postal surveys & qualitative interviews	Specialist supportive workers were effective in enabling family carers to realise EoL care in the home, through the provision of practical assistance and emotional support and reassurance.	32
**Sterling et al (2018) USA [27]**	46	Community setting	Homecare workers	To explore the perspectives of workers who care for adults with heart failure	Focus groups	Diagnosis-specific training is not given, condition changes can be challenging for staff: additional training would add confidence and skills.	34
**Turnbull et al (2020) Honk Kong & Australia [39]**	9	Community setting	Homecare workers	To study of the impact of the COVID-19 crisis on the communication practices of EoL care workers	Mixed method of survey and interview	The findings of this study highlight the fundamental importance of both verbal and non-verbal communication to the relationships established between non-clinical workers and service users.	33

**Wladkowski et al (2021) USA [28]**	24	Community setting	Direct care workers	To explore workers' experiences of loss and grief over their clients	Focus groups & individual interviews	Increased organizational support and training could help address grief and loss.	35
**Watanabe et al (2013) Japan [34]**	458 (371 female)	Community setting	Care managers	To explore care managers' confidence in managing home-based EoL care	Questionnaires	Increased confidence associated with previous experience or nursing qualifications: training could increase confidence in those without this background.	35
**Yamamoto -Mitani et al (2015) Japan [35]**	1,159	Community settings	Home care nurses, home helpers, and care managers	To explore participants' experiences in home-based EoL	Questionnaires	Collaboration could be improved by understanding roles of other professionals and better communication.	33
**Yeh et al (2019) England [29]**	42	Community setting	Home care works & managers	To enhance understanding about workers providing care to people with dementia at EoL	Semi-structured interview	Peer and manager support are essential and effective in coping with pressures. Home care work is isolating increasing risk of stress going unnoticed.	34

* Based on Hawker et al., [21]. Reviewed by third assessor if two assessors in disagreement.

EoL: End of life

Online Supplemental Table 2. Quality Appraisal

Author (date) Country	Abstract Title	Introduction Aims	Method Data	Sampling	Data analysis	Ethics Bias	Findings Results	Transferability Generalisability	Implications Usefulness
Abrams et al (2018) UK [5]	4	3	4	4	4	4	4	4	4
Baik et al (2021) USA [36]	4	4	4	4	4	4	4	4	4
Breen et al (2021) Canada [23]	4	4	4	3	4	4	4	4	4
Craftman et al (2022) Sweden [30]	4	4	4	3	4	4	4	3	4
Fujita et al (2019) Japan [31]	4	3	3	3	4	3	4	3	3
Fukui et al (2019) Japan [32]	4	4	4	3	4	3	4	3	4
Igarashi et al (2015) Japan [33]	4	4	3	4	4	3	4	3	3
Lee et al (2017) UK [24]	4	4	4	3	3	3	4	4	4
Manson et al (2020) England [15]	4	4	3	3	3	3	3	3	3

**McPherson et al (2019) Canada [37]**	4	4	4	3	4	4	4	4	4
**Odierna et al (2018) USA [25]**	4	4	4	4	4	3	4	4	4
**Percival et al (2013) UK [26]**	4	4	4	4	4	3	4	3	3
**Poulos et al (2017) Australia [38]**	4	3	3	4	3	3	4	4	4
**Sterling et al (2018) USA [27]**	4	4	4	3	4	4	4	3	4
**Turnbull et al (2020) Honk Kong & Australia [39]**	4	4	4	3	4	3	4	4	3
**Wladkowski et al (2021) USA [28]**	4	4	4	4	4	3	4	4	4
**Watanabe et al (2013) Japan [34]**	4	4	4	4	4	3	4	4	4
**Yamamoto-Mitani et al (2015) Japan [35]**	4	4	3	4	4	4	4	3	3
**Yeh et al (2019) England [29]**	4	4	4	4	3	3	4	4	4
